# Survival analyses of the ZeOxaNMulti trial: Follow-up randomized, double-blinded, placebo-controlled trial of oral PMA-zeolite to prevent chemotherapy-induced side effects, especially peripheral neuropathy

**DOI:** 10.3389/fphar.2022.874028

**Published:** 2022-11-08

**Authors:** Maria Giuseppa Vitale, Anna Crispo, Dario Arundine, Riccardo Ronga, Carmela Barbato, Assunta Luongo, Francesco Habetswallner, Bernardo Maria De Martino, Angela Maione, Sandra Eisenwagen, Giovanna Vitale, Ferdinando Riccardi

**Affiliations:** ^1^ Medical Oncology Unit, University Hospital of Modena, Modena, Italy; ^2^ Epidemiology and Biostatistics Unit, Istituto Nazionale Tumori-IRCCS “Fondazione G. Pascale”, Naples, Italy; ^3^ Medical Oncology Unit, AORN Antonio Cardarelli, Naples, Italy; ^4^ UOC Neurofisiopatologia AORN Cardarelli, Naples, Italy; ^5^ Panaceo International GmbH, Villach, Austria; ^6^ School of Medicine and Surgery, University of Campania Luigi Vanvitelli, Caserta, Italy

**Keywords:** oxaliplatin, PMA-zeolite, chemotherapy-induced peripheral neuropathy, colorectal cancer, disease-free survival, progression-free survival, overall survival, follow-up

## Abstract

Following the previously published results of the clinical randomized ZeOxaNMulti trial, we evaluated the potential of the tested product PMA-ZEO (Multizeo Med) in the prevention of chemotherapy-induced side effects (especially peripheral neuropathy) within a 30-month follow-up analysis. The aim was to determine the disease-free survival (DFS), progression-free survival (PFS), and overall survival (OS) in a study-population suffering from colorectal cancer that was previously enrolled in the ZeOxaNMulti trial from April 2015 to October 2018. The participants of the study were randomized to receive either PMA-ZEO or placebo while undergoing oxaliplatin-based chemotherapy. A total of 104 patients (pts) (51% of participants randomized to the PMA-ZEO group and 49% to the placebo group), out of a total of 120 pts included in the ZeOxaNMulti trial in 2015, were followed up until March 2021 and were included in the follow-up analysis. According to the chemotherapy line, 44.2% of patients received chemotherapy in an adjuvant setting, and 55.8% of patients received chemotherapy as first-line treatment. The statistical analysis for DFS, PFS, and OS was performed by comparison of the end results with data from the PMA-ZEO/placebo-intervention start point. The analysis of OS did not show statistically significant differences in the first-line chemotherapy patients randomized to PMA-ZEO than among the placebo group (*p* = 0.1) over the whole period of follow-up (30 months). However, focusing on the PMA-ZEO supplementation time point (7 months), a positive and statistically significant trend (*p* = 0.004) was documented in the OS analysis for the first-line chemotherapy patients with increasing months of PMA-ZEO treatment compared to the placebo group. Furthermore, borderline statistical significance was reached for PFS at the PMA-ZEO supplementation time point (7 months) in the first-line chemotherapy patients (*p* = 0.05) for cancer progression events. After stratification of the first-line chemotherapy patients, statistically relevant trends for OS for age, comorbidities, and oxaliplatin dosage (cycles) were also determined. The overall results for DFS (adjuvant patients), PFS (first-line chemotherapy patients), and OS (adjuvant and first-line chemotherapy patients) were generally slightly better in the PMA-ZEO group than in the placebo group, even though no statistically significant results were obtained between the groups within the follow-up period until 2021 (30 months). Based on this follow-up analysis, protective effects of PMA-zeolite supplementation can be deduced. A positive trend and more importantly, significant results in PFS and OS for specific patient groups during and/or after PMA-ZEO treatment were determined, which supports the use of PMA-ZEO as an oncological supportive therapy.

## Introduction

According to GLOBOCAN statistics, colorectal cancer remains the third most commonly diagnosed cancer and the second leading cause of cancer deaths worldwide, accounting for 10.2% of newly diagnosed cancer and 9.2% of cancer-related deaths (Bray et al., 2018). Chemotherapy treatment with FOLFOX (5-fluorouracil/leucovorin and oxaliplatin) and FOLFIRI (5-fluorouracil/leucovorin and irinotecan) schemes have been established as efficacious regimens for improving the overall survival (OS) in adjuvant and in metastatic patients (Gustavsson et al., 2015). Additionally, several side effects related to oxaliplatin therapy were reported (Cassidy and Misset, 2002), including hematological and gastrointestinal tract toxicity and, in particular, peripheral neuropathy. These side effects are due to the inability of chemotherapeutic agents to differentiate between healthy and malignant cells. Zeolites (clinoptilolite materials) are naturally occurring aluminosilicates, mainly of volcanic origin. They have a microporous network forming small individual channels and cavities possessing cation-exchange capacity. Recently, the application of a specific natural zeolite material, that is, the Panaceo-Micro-Activation (PMA)-zeolite (clinoptilolite material) (PMA-ZEO), has been proven to be an efficient and safe option for many medical purposes as well as oral application in humans. The plausible positive impact induced by the specific PMA-ZEO remains the same as in our previous study (Vitale et al., 2020). The ZeOxaNMulti trial, a randomized, double-blinded controlled trial based on oral PMA-ZEO administration in cancer patients (pts), aimed to prevent chemotherapy-induced side effects in 120 pts predominantly diagnosed with colorectal cancer requiring oxaliplatin-based chemotherapy. The patients were randomized to receive either PMA-ZEO (Multizeo Med) or placebo (microcrystalline celluloses) while undergoing oxaliplatin-based chemotherapy (FOLFOX or XELOX scheme). A nerve-conduction study (NCS) was planned at baseline and at the conclusion of three and 6 months of chemotherapy with the aim of evaluating chemotherapy-induced peripheral neuropathy (CIPN). CIPN is indeed the most frequently reported adverse effect of oxaliplatin therapy. Furthermore, the evaluation of hematological and liver toxicity was performed during every cycle of chemotherapy. The group treated with PMA-ZEO showed a lower CIPN (although not statistically significant for the whole group of pts) compared to pts receiving placebo. This advantage was, however, statistically significant in the male subgroup (*p* = 0.047). In addition, supplementation with the PMA-ZEO resulted in a lower incidence of severe-grade hematological toxicity, although full statistical significance was not achieved (*p* = 0.09). Due to the low liver toxicity incidence, statistical analysis was not performed. Patients treated with PMA-ZEO were able to undergo more cycles of chemotherapy (*p* = 0.03), which also indicates a significant improvement in tolerance to the therapy (Vitale et al., 2020). Overall, there was a better tolerability of chemotherapy (increase in cycles) enabling better adherence to the oncology treatment protocol in pts treated with PMA-ZEO in comparison with placebo.

In addition, the PMA-ZEO clinoptilolite has already been tested *in vitro* and *in vivo* in animals and humans, providing useful information to evaluate its adjuvant effects in specific medical conditions, that is, cancer (Eisenwagen, 2020). The research or application of appropriate zeolite-clinoptilolite products as adjuvant therapy to standard therapies in human medicine is a growing field. This is due to the previously studied zeolite-clinoptilolite anti-oxidant effect (Dogliotti et al., 2012), hemostatic characteristics (Pavelic et al., 2001), anti-diarrheic properties (Rodríguez-Fuentes et al., 1997), or immunomodulatory properties (Ivkovic et al., 2004). Finally, a recent study on human subjects showed that oral supplementation with PMA-ZEO reduces inflammation in the gut of irritable bowel syndrome (IBS) patients and has a positive impact on the intestinal microbiota (Petkov et al., 2021).

A possible explanation of the PMA-ZEO positive effects in an adjuvant therapeutic regimen is, accordingly, based on its main site of action in the gastrointestinal tract that results in detoxification of components involved in the development of neuropathy (e.g., ammonium—recognized as a neurotoxic agent produced by tumors), as well as on positive impact on immunity and oxidative stress.

These effects of PMA-ZEO could be attributed to its porous structure, which has the ability to capture ions and molecules into its holes (Mastinu et al., 2019). The mechanism of action in the gastrointestinal tract is quite specific as the negatively charged channels and cavities are occupied with positively charged alkali, and alkali earth monovalent (i.e., Na+, K+) and divalent (i.e., Ca2+) ions, OH-groups, or H_2_O molecules. These molecules are immediately exchangeable with other elements and cations from the surrounding environment in the gastrointestinal tract. Along with the release of positively charged minerals, other molecules and cationic groups from the surroundings, such as ammonia, can be accommodated inside the porous structure (Margeta et al., 2013; Gaikwad and Warade, 2014). The cations are bound to the zeolite (clinoptilolite material) based on their selectivity alignment (Kraljevic Pavelic et al., 2018). In addition to ammonia, which is generally known as a neurotoxin and is produced by tumors or during chemotherapy, numerous heavy metals, such as Pb, As, Cr, Ni, or Cd, may be exchanged under the physiological conditions of the gastrointestinal tract (Kraljević Pavelić et al., 2017). However, the zeolite (clinoptilolite material) ion-exchange effects *in vivo* are quite complex and depend on both the environmental conditions (pH, temperature, *etc.*) and material composition/cation affinity properties and are, therefore, not linearly explainable. Another essential issue that determines the ion-exchange capacity and attraction of cations is the final Si/Al ratio in the clinoptilolite material (Kraljevic Pavelic et al., 2018) (Mumpton, 1999).

The positive systemic mechanism of natural zeolites (clinoptilolite materials) is yet to be fully elucidated. Pavelić et al. hypothesized that the effects of natural zeolites may be at least partially attributed to the restoration of human homeostasis due to local detoxification properties within the intestine, the release of dissolved silica forms from the clinoptilolite-tuff that enters from the intestine into the blood, and clinoptilolite’s immunomodulatory effects involving the induction of immune responses through Peyer’s patches and/or possible positive effects on microbial intestinal populations. These local effects may strengthen the whole immune system (Kraljevic Pavelic et al., 2018). Importantly, there seems to be a correlation between the diversity of the gut bacteria and the response to cancer therapy—specifically in the case of immunotherapy (Shui et al., 2019). Furthermore, it is possible that drugs used in cancer therapy might have a negative impact on the gastrointestinal wall through severe negative changes in the microbiota population (Farhadi et al., 2003). In various studies, it was shown that PMA-zeolite has a positive effect on the gastrointestinal wall (Petkov et al., 2021; Kraljevic Pavelic et al., 2022). This positive effect may be hypothesized in cancer patients, especially considering significant adverse effects in cancer patients treated with diverse cancer drugs. Approximately ¼ of cancer patients treated with these drugs report impaired quality of life, whereby gastrointestinal symptoms are the most commonly reported side effects and are most strongly associated with a decreased quality of life (O'Reilly et al., 2020)**.** Studies suggest the use of prebiotics as a combined therapy approach for patients with colorectal cancer (Ding et al., 2018). Moreover, a correlation between a healthy state of the gut and the diversity of gut bacteria, in particular, has been established as a requirement for a proper response to cancer therapy, specifically immunotherapy (Shui et al., 2019).

Furthermore, while the acute CIPN induced by oxaliplatin is due to the temporary interaction with voltage-gated sodium channels (Na+) in the nerve membrane, PMA-ZEO might have a positive impact due to the release of sodium (Na+) from its structure during the ion-exchange process. As Na+ is readily exchangeable from clinoptilolite material, its levels and absorption in the body should not be altered (Vitale et al., 2020).

Based on these previously demonstrated positive effects, we conducted a follow-up analysis of the ZeOxaNMulti trial to specifically determine the safety and benefits of PMA-ZEO as oncologically supportive therapy through the evaluation of disease-free survival (DFS), progression-free survival (PFS), and overall survival (OS) of patients diagnosed with colorectal cancer, who were either adjuvantly treated with PMA-ZEO or with placebo (microcrystalline cellulose). The analysis of DFS, PFS, and OS were performed for the follow-up period of 30 months and for the PMA-ZEO treatment end point (7 months).

## Patients and methods

### Patients

From April 2015 to October 2018, 120 pts predominantly diagnosed with colorectal cancer (*n* = 112) or other cancer types (*n* = 8) from the Oncology Department, Antonio Cardarelli Hospital, Naples, Italy, were screened for eligibility and enrolled in the ZeOxanMulti trial. From 120 pts previously enrolled in the original study, 109 patients completed the 30-month follow-up period until March 2021. Among these 109 patients, the participants with other cancer diagnosis (*n* = 5) were excluded from the follow-up analysis, resulting in a final sample size of 104 patients diagnosed with colorectal cancer.

The inclusion criteria were a histologically confirmed diagnosis of colon cancer, at least 18 years of age, oxaliplatin chemotherapy, and adequate hematologic parameters to allow chemotherapy. Only patients treated with oxaliplatin, as a known neurotoxic cytostatic, were included. The exclusion criteria were chemotherapy treatment with neurotoxic drugs (cis-platin, carboplatin, oxaliplatin, vincristine, vinblastine, paclitaxel, or docetaxel) in the 6 months prior to the start of oxaliplatin-chemotherapy, pregnancy, or breastfeeding. All participants gave their written informed consent.

Eligible patients were randomized 1:1 into two groups: the PMA-ZEO and placebo groups. Further details relating to the supplementation are contained in our previous study (Vitale et al., 2020). Briefly, the supplementation was carried out according to the following scheme: the experimental group received 6 g/day PMA-ZEO (Multizeo Med, Goedersdorf, Austria) in two daily doses of 3 g, while the placebo group received 6 g/day placebo (microcrystalline cellulose as it was used in another randomized, double-blinded, controlled trial) in two daily doses of 3 g. The placebo and experimental treatments were indistinguishable in size, weight, and their characteristics.

### Materials and Methods

The study was approved by the ethical standards of the Antonio Cardarelli Hospital Ethical Committee (protocol number 107 of 19/02/2015). This study was conducted according to the guidelines of the Declaration of Helsinki for Research on Human Subjects 1989.

The PMA-ZEO (natural clinoptilolite material) (Multizeo Med, Goedersdorf, Austria) used for this study is an approved certified medical device for human use throughout Europe, micronized by the use of a patented method (patent WO 2018/100178A1). Due to the changes in the biophysical properties based on the patented micronization process (Panaceo-Micro-Activation - PMA), a generalization of the presented outcomes to other clinoptilolite materials would require additional clinical studies, as previous findings demonstrate that the Panaceo-Micro-Activation (PMA) changes the biophysical properties of the clinoptilolite used (patent WO 2018/100178A1). PMA-ZEO has been previously studied by the use of required toxicology tests according to the ISO standards, OECD guidelines, and several other clinical studies (Petkov et al., 2021) (Lamprecht et al., 2015; Kraljevic Pavelic et al., 2022).

A total of 49 pts received oxaliplatin in an adjuvant setting, 60 pts in first-line, 7 pts in second-line, and 3 pts in third-line of treatment (no information about the treatment setting was available for one patient). The DFS, PFS, and OS were statistically analyzed at follow-up of 30 months considering the period of PMA-ZEO intake of 7 months as well, in particular for PFS and OS. Information regarding sex, age, comorbidities, and treatment setting were recorded as possible confounding factors. Data on the clinical condition and information on the staging of disease were collected by physicians during medical control visits, using diagnostic imaging and physical examination, whose specific timing depends on the treatment setting of each patient enrolled in the trial. Two medical control visits were scheduled for month 3 and 6 during the 2 years of follow-up. For metastatic patients in treatment of first-, second-, or third-line of therapy, medical control visits of re-evaluation were planned for each quarter of the year.

### Statistical analyisis

Descriptive statistics for the categorical data was reported. Disease-free survival (DFS) as an endpoint for patients (*n* = 49) undergoing chemotherapy in an adjuvant setting (adjuvant patients) was defined as the time between starting PMA-ZEO and recurrence for adjuvant therapy. Progression-free survival (PFS) was defined as the time between starting PMA-ZEO and recurrence for the first-line therapy. PFS was estimated for patients undergoing chemotherapy in the first-line treatment (first-line chemotherapy patients) (*n* = 60). Overall survival (OS) was defined as the time from starting PMA-ZEO to death from any cause. Log-rank tests (DFS, PFS, and OS) were used to compare survival endpoints in the two arms. Hazard ratios (HRs) and the corresponding 95% confidence interval (CI) were estimated using a Cox model. Statistical analyses were performed using the Statistical Package for Social Science (SPSS), statistical software version 26 (SPSS Inc., Chicago, IL, United States). Post-hoc subgroup analysis was performed; the subgroups based on sex, age, comorbidities, dose reduction, and number of chemotherapy cycles were precisely predefined according to their subgroup categories (respectively, <60 years, 61–65 years, 66–70 years, and ≥70 years for age; yes or no for dose reduction and comorbidity; and <9 cycles and ≥9 cycles for chemotherapy).

### Sample size calculation

The power calculation of the study was based on the primary objective—the reduction of the acute CIPN in patients treated with oxaliplatin. Assuming an incidence of acute CIPN of 85% among patients treated with oxaliplatin-based chemotherapy, our study has a power of 86% to show that the acute CIPN incidence rate in the PMA-ZEO group is 75%. Assuming that the difference is 10% points or less between the two groups and its 95% confidence interval, as well as that alpha (2-tailed) is set at 0.05, the sample included two groups with 60 pts each. Formally, there was an 86.5% probability that the 95% confidence interval for the difference in event rates would exclude a 10%-point difference in favor of the placebo group.

## Results

### Demographic characteristics

Among 120 pts included in the ZeOxaNMulti trial in 2015 receiving either PMA-ZEO or placebo, a total of 109 pts were followed-up until March 2021. Among these, pts with different cancer diagnoses (*n* = 5) were excluded from the follow-up analysis, leaving a final sample size of 104 pts with a median follow-up duration of 39.4 months (range: 7–74 months). The characteristics of the participants are shown in Table 1. Among 104 pts, 51% were randomized to the PMA-ZEO group and 49% to the placebo group. The majority of participants were male (51.9%) and under 60 years of age (40.4%). A total of 44.2% patients received chemotherapy in an adjuvant setting, and 55.8% patients received chemotherapy as the first-line of treatment.

**TABLE 1:** Sample characteristics.

	*N* = 104	%
Sex		
Male	54	(51.9)
Female	50	(48.1)
Age		
<60	42	(40.4)
61–65	12	(11.5)
66–70	27	(26.0)
>70	23	(22.1)
Chemotherapy line		
Adjuvant	46	(44.2)
First-line	58	(55.8)
Arm of intervention		
Placebo	51	(49)
PMA-ZEO	53	(51)

### Univariate analysis for DFS, PFS, and OS for the follow-up period of 30 months

**FIGURE 1:**
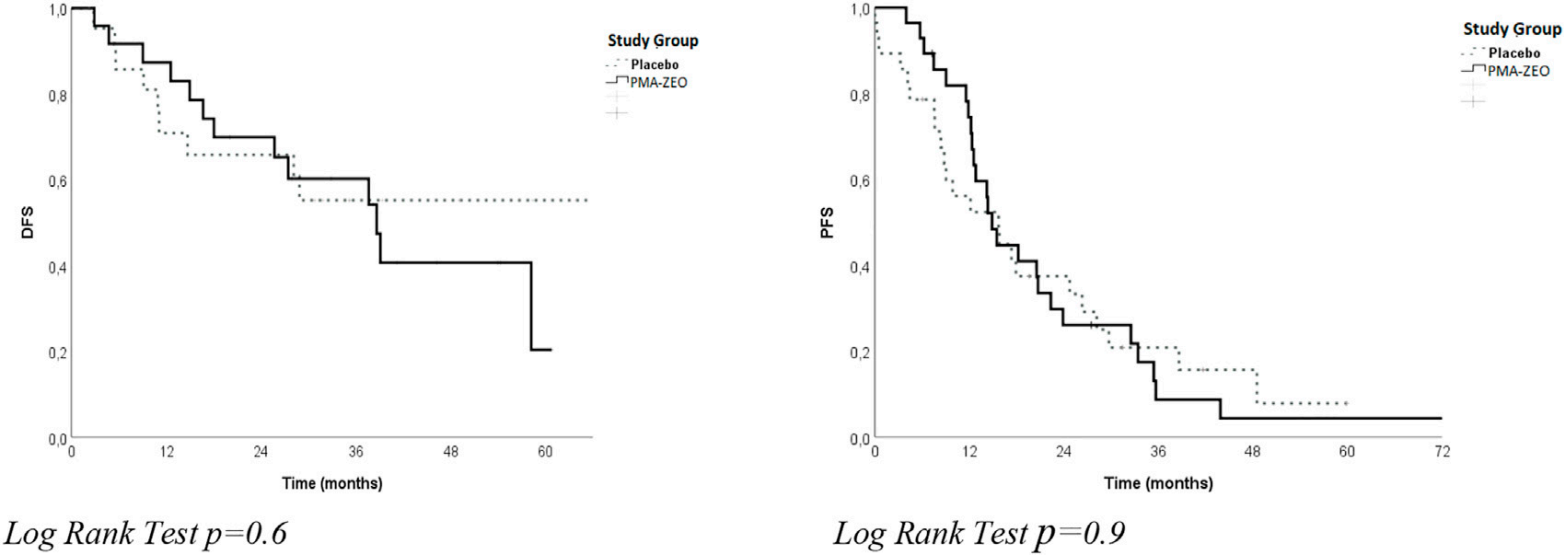
Disease-free survival (DFS) and progression-free survival (PFS) for the study groups (Placebo vs. PMA-ZEO).

The analysis of overall DFS and overall PFS was performed for adjuvant patients and for first-line chemotherapy patients, respectively. The overall DFS was not significantly different between the groups (median time of DFS = 39.1 months, CI = 15.3–62.9, *p* = 0.6). Similarly, the overall PFS was not significantly different between the groups (median time of PFS = 15.5 months, CI = 11.9–19.0 *p* = 0.9) (Table 2; Figure 1).

**TABLE 2:** Univariate analysis for DFS, PFS, and OS for the follow-up period of 30 months.

	Disease-free survival (DFS)					Progression-free survival (PFS)				
	Adjuvant					First-line				
	N° events/Total		Median (95% CI)		*p*-value**	N° events/Total		Median (95% CI)		*p*-value^**^
PMA-ZEO treat. group overall	22/46		39.1 (15.3–62.9)		0.6	49/56		15.5 (11.9–19.0)		0.9
	Placebo	PMA-ZEO	Placebo	PMA-ZEO		Placebo	PMA-ZEO	Placebo	PMA-ZEO	
	9/22	13/24	n.e.	38.6 (25.2–57.1)		23/28	26/28	15.7 (5.7–25.7)	14.9 (12.7–7.0)	
Overall survival (OS)										
	Adjuvant					First-line				
	N° events/Total		Median (95% CI)		p-value**	N° events/Total		Median (95% CI)		*p*-value^**^
PMA-ZEO treat. group overall	14/46		58.6 (54.7–62.6)			39/56		30.2 (22.6–37.9)		
	Placebo	PMA-ZEO	Placebo	PMA-ZEO		Placebo	PMA-ZEO	Placebo	PMA-ZEO	
	7/22	7/24	56.7 (n.e.)	58.7 (31.5–85.8)	0.8	22/28	17/28	26.3 (16.6–36.1)	37.1 (31.1–43.1)	0.1
Age										
*<60 years*	2/7	3/12	56.7 (n.e)	n.e.	0.9	8/7	8/14	16.5 (0.01–39.1)	37.1 (23.4–50.8)	0.09
*61–65 years*	1/4	1/1	35.0 (n.e.)	4.5 (n.e.)	0.05	3/5	1/2	40.6 (0.01–90.1)	15.5 (n.e.)	0.7
*66–70 years*	3/7	1/7	n.e.	n.e.	0.3	3/6	4/6	59.9 (n.e.)	17.8 (0.01–37.7)	0.3
*>70 years*	1/4	2/4	n.e.	58.7 (n.e.)	0.7	9/9	4/6	21.8 (15.2–28.3)	39.7 (3.1–76.3)	0.06
Sex										
*Male*	4/12	5/15	56.7 (32.4–80.9)	n.e.	0.9	13/17	8/10	25.0 (3.3–46.7)	26.3 (3.6–48.9)	0.7
*Female*	3/10	2/9	n.r.	58.7 (n.e.)	0.8	9/11	9/18	28.5 (17.8–39.1)	37.1 (30.1–44.1)	0.3
*N° of cycles*										
*<9 cycles*	2/9	1/6	n.e.	n.e.	0.6	8/10	2/6	10.7 (0.9–20.4)	n.e.	0.06
*≥9 cycles*	5/13	6/18	56.7 (39.9–73.4)	58.7 (n.e.)	0.9	14/18	15/22	30.2 (26.7–33.6)	37.1 (16.7–57.2)	0.8
Dose reduction										
*No*	4/10	5/9	56.8 (n.e.)	30.6 (16.7–44.5)	0.2	12/16	6/12	21.8 (4.3–39.3)	37.5 (24.6–50.5)	0.2
*Yes*	3/10	2/15	n.e.	58.7 (n.e.)	0.3	10/12	11/16	26.4 (18.9–33.9)	37.2 (16.2–58.1)	0.6
Comorbidity										
*No*	3/7	4/14	56.7 (24.5–88.9)	58.7 (n.e.)	0.8	10/11	8/14	25.0 (2.8–47.1)	37.1 (14.4–59.8)	0.1
*Yes*	4/15	3/10	n.e.	n.e.	0.9	12/17	9/14	28.5 (12.2–44.7)	33.9 (16.7–51.0)	0.7

**Log-rank test.

**FIGURE 2:**
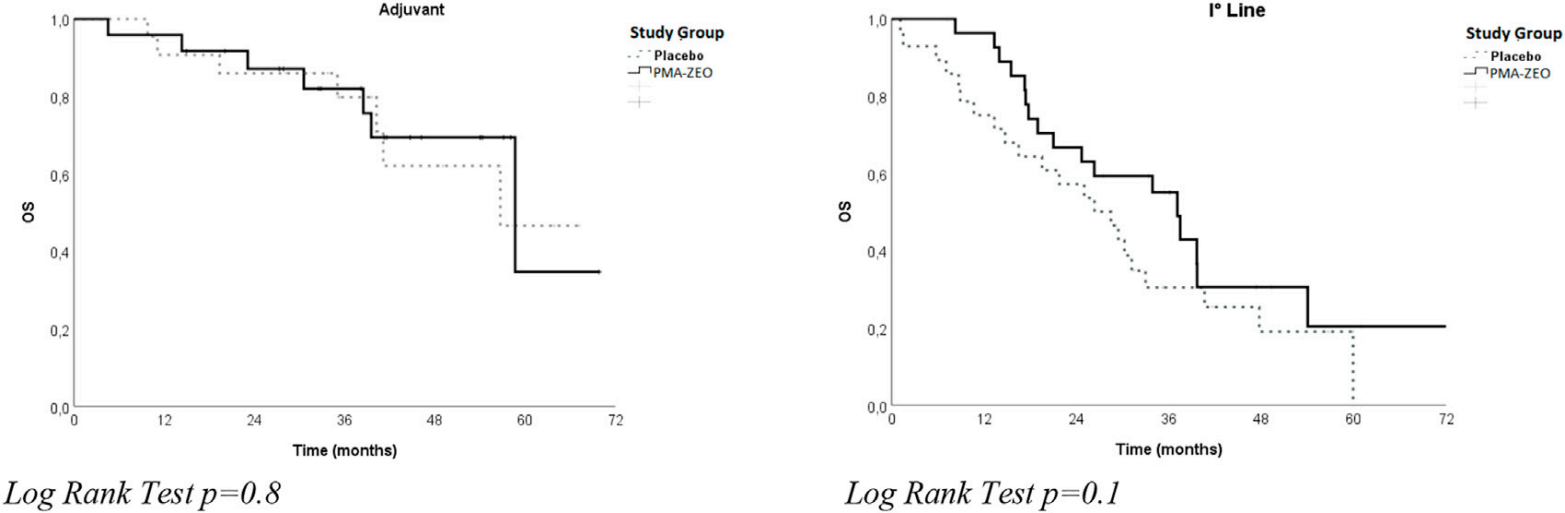
Overall survival (OS) for study groups (Placebo vs. PMA-ZEO) stratified by adjuvant and I° line treatment.

The analysis of OS was performed both for adjuvant patients and for first-line chemotherapy patients in the PMA-ZEO and placebo groups. In particular, a longer OS was found in the first-line chemotherapy patients for the PMA-ZEO group than among the placebo group (median time of OS = 37.1 months, CI = 31.1–43.1 vs. median time of OS = 26.3 months, CI = 16.6–36.1); however, this difference was not significant (*p*-value = 0.1). Additionally, among adjuvant patients, the OS in the PMA-ZEO group was nearly the same as that in the placebo group (median time of OS = 58.7 months, CI = 31.5–85.5 vs. median time of OS = 56.7 months, CI = ne, *p*-value = 0.8) (Table 2; Figure 2).

The results of OS analysis stratified for age, sex, number of chemotherapy cycles, oxaliplatin dose reduction, and comorbidity are shown in Table 2. After stratification for age, a longer OS was found among younger (age<60 years) first-line chemotherapy patients in the PMA-ZEO group (median time of OS = 37.1 months, CI = 23.4–50.8) than among the placebo group (median time of OS = 16.5 months, CI = 0.01–39.1) with a positive trend (*p* = 0.09). A longer OS was observed in elderly patients (age>70 years) and first-line chemotherapy pts in the PMA-ZEO group (median time of OS = 39.7 months, CI = 3.1–76.3) in comparison with the placebo group (median time of OS = 21.8 months, CI = 15.2–28.3); the difference was marginally significant (*p* = 0.06).

**FIGURE 3:**
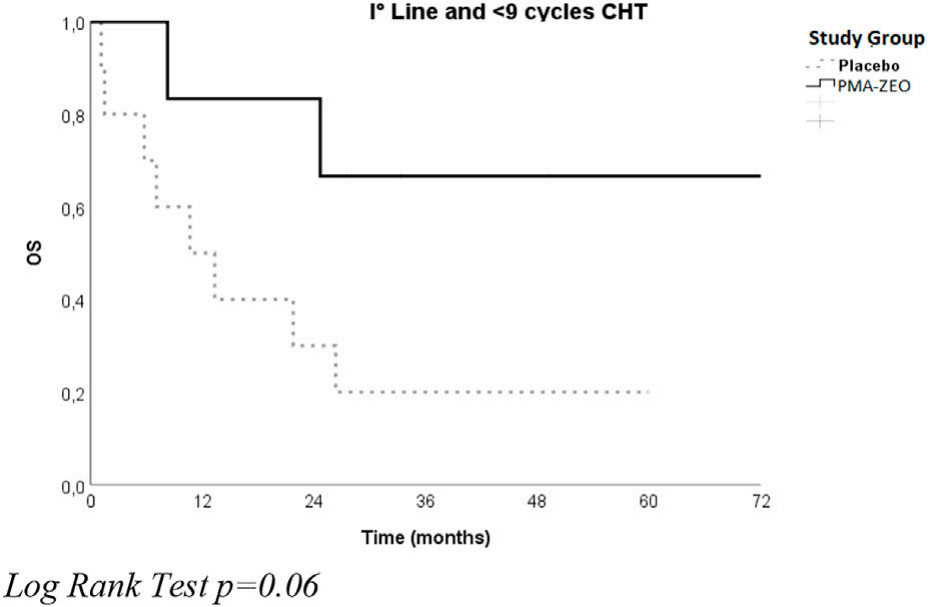
Overall survival for the study groups (Placebo vs. PMA-ZEO) stratified by I° line treatment and <9 cycles of chemotherapy (CHT).

After stratification for sex, there was no significant difference in OS between the placebo group and the PMA-ZEO group, particularly for men who underwent first-line chemotherapy (median time of OS = 26.3, CI = 3.6–48.9 vs. median time of OS = 25.0, CI = 3.3–46.7) (*p*-value = 0.7). However, after stratification for the number of chemotherapy cycles, a longer OS was found for the first-line chemotherapy pts in the PMA-ZEO group that were able to undergo more chemotherapy cycles (>9 cycles) (median time of OS = 37.1, CI = 16,7–57.2) than among the placebo group (median time of OS = 30.2, CI = 26.7–33.6); the difference was not statistically significant (*p* = 0.8) (Figure 3).

Additionally, after stratification for oxaliplatin dose reduction, a longer OS was found for the first-line chemotherapy patients in the PMA-ZEO group (*p* = 0.2) that did not reduce the oxaliplatin dose (median time of OS = 37.5 months, CI = 24.6–50.5) compared to the placebo group (median time of OS = 21.8 months, CI = 4.3–39.3). Furthermore, after stratification for comorbidity, a longer OS was found for first-line chemotherapy patients with no comorbidities in the PMA-ZEO group than among the placebo group (median time of OS = 37.1, CI = 14.4–59.8 vs. median time of OS = 25.0, CI = 2.8–47.1) although these results are not statistically significant (*p* = 0.1).

### Univariate analysis for PFS and OS at the PMA-ZEO supplementation at the 7-month time-point

**FIGURE 4:**
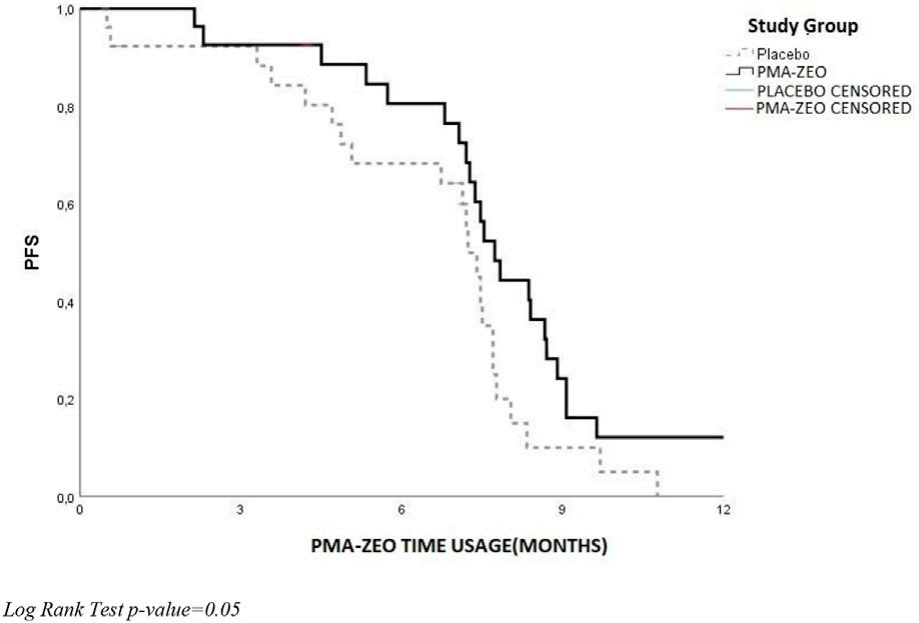
Progression-free survival (PFS) at the PMA-ZEO 7-month time point for cancer relapse stratified by I° line treatment group.

**FIGURE 5:**
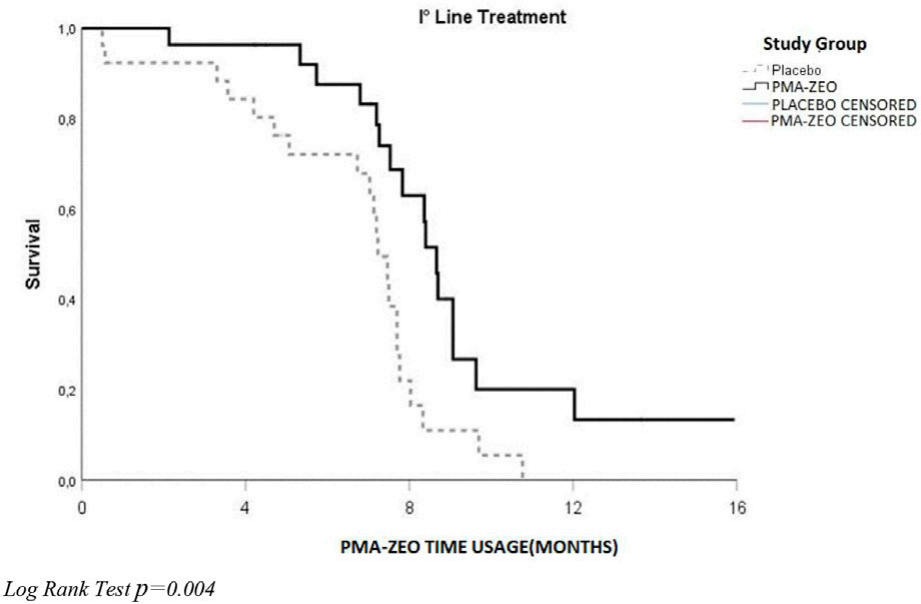
Overall survival (OS) at the PMA-ZEO 7-month time point for death events stratified by I° line treatment group.

The analysis of PFS and OS was performed at the 7-month time-point of the PMA-ZEO supplementation for first-line chemotherapy patients randomized to the PMA-ZEO or placebo group. There was a marginally significant difference in PFS (*p*-value = 0.05), although the median PFS time did not differ between the PMA-ZEO and placebo groups (median time of PFS = 7.73, CI = 6.83–9.13 vs. median time of PFS = 7.23, CI = 5.48–7.48) (Table 3; Figure 4). However, a significantly longer OS was observed in the PMA-ZEO group with increasing months of PMA-ZEO supplementation than among the placebo group (median time of OS = 8.67, CI = 8.23–9.10, vs. median time of OS = 7.23, CI = 6.78–7.68, *p* = 0.004) (Table 4; Figure 5).

**TABLE 3:** PFS at the PMA-ZEO 7-month time point for cancer progression events in first-line treatment patients.

Progression-free survival	N°events/total	Median time (95% CI)	*p*-value
Groups	—	—	0.05
Placebo group	22/27	7.23 (5.48–7.48)	—
PMA-ZEO	25/27	7.73 (6.83–9.13)	—

**TABLE 4:** OS at the PMA-ZEO 7-month time point for death events in first-line treatment patients.

Overall survival	N°events/total	Median time (95% CI)	*p*-value
Groups	—	—	0.004
Placebo group	21/26	7.23 (6.78–7.68)	—
PMA-ZEO	16/27	8.67 (8.23–9.10)	—

### Multivariate cox regression analysis

The results of univariate survival analysis showed that first**-**line chemotherapy patients had measurable benefits from PMA-ZEO treatment in comparison with the placebo group, particularly for OS. Therefore, multivariate Cox regression analysis was performed for first-line chemotherapy patients in the PMA-ZEO group. This multivariate analysis (adjusted for age, sex, and number of chemotherapy cycles) revealed protective effects of PMA-ZEO supplementation in terms of survival (HR = 0.57, CI = 0.29–1.13) although this result was not statistically significant (*p*-value = 0.10) (Table 5).

**TABLE 5:** Adjusted multivariate Cox regression analysis for the first-line treatment group.

	HR^a^	95% CI	*p*-value
Groups			
Placebo	1^b^	—	—
PMA-ZEO	0.57	0.29–1.13	0.10

^a^ Adjusted for age, sex, and number of cycles.

^b^ Reference category.

## Discussion

Zeolites, especially the clinoptilolite material examined herein, are naturally occurring aluminosilicates of volcanic origin. Recently, the application of a specific natural zeolite-clinoptilolite material, Panaceo-Micro-Activation (PMA)-zeolite, has been proven to be an efficient and safe option for clinical use. The PMA-zeolite (PMA-ZEO) is a certified medical device characterized by detoxifying, anti-oxidant, and anti-inflammatory properties (Vitale et al., 2020).

In particular, growing evidence derived from *in vitro* and *in vivo* studies suggests that zeolite-clinoptilolite might have a direct effect on tumors, in particular influencing key molecules involved in metastasis, cell viability and survival, division, and stress response (Eisenwagen, 2020). Recently, it was shown that zeolite in the nanoparticulate form (nano-clinoptilolite) decreased cell viability and induced caspase-3 and 7, thereby mediating apoptosis in osteosarcoma dog cell lines. Additionally, the nano-clinoptilolite form increased BAX/BCL-2 ratio, thus activating mitochondrial apoptosis (Ulutaş et al., 2020).

In this study, we present a continuation of our previous studies on cancer patients supplemented with PMA-ZEO as a 30-month follow-up analysis (Vitale et al., 2020). The present study evaluated the effects of PMA-ZEO survival in patients diagnosed with colorectal cancer previously enrolled in the ZeOxaNMulti trial. Subgroup analysis was obtained upon stratification for sex, age, comorbidities, number of chemotherapy cycles, and dose reduction.

DFS analysis was considered for adjuvant patients, PFS was calculated for first**-**line chemotherapy patients, and OS was calculated separately for adjuvant and first**-**line chemotherapy patients. These particular groups were randomized into the PMA-ZEO or placebo groups. Univariate analysis did not show statistically significant differences between the PMA-ZEO and placebo groups in terms of DFS (for adjuvant patients) or PFS (for first**-**line chemotherapy patients). However, OS analysis showed that PMA-ZEO exhibited greater beneficial effects on first**-**line chemotherapy patients than adjuvant patients; specifically, younger (age<60 years) and elderly (age>70 years) first**-**line chemotherapy patients had a longer OS than the placebo group. Additionally, protective effects of PMA-ZEO were observed in the first**-**line chemotherapy patients with no comorbidities. In contrast, sex or dose chemotherapy reduction did not affect OS analysis, both in either adjuvant or first**-**line chemotherapy patients. These positive results for OS were even stronger in the analysis for the 7-month PMA-ZEO supplementation time point in first**-**line chemotherapy patients: a significantly longer OS was observed for the PMA-ZEO group (*p* = 0.004). Furthermore, PFS analysis indicated that there was a borderline significant difference between the study groups when the time of PMA-ZEO usage was evaluated (*p* = 0,05). The results of the univariate survival analysis were confirmed by multivariate Cox regression analysis, which was performed only for first**-**line chemotherapy patients randomized to the PMA-ZEO or placebo group. This analysis particularly highlighted the protective role of PMA-ZEO in the survival rate.

Borderline statistically significant differences and trends, or in specific cases, statistical significance was identified between the PMA-ZEO and placebo groups during the 7-month supplementation period for OS and PFS for the first**-**line chemotherapy patients. These results could be explained by the previously described correlation between PMA-ZEO anti-cancer activity, material anti-inflammatory, and immune-modulatory effects. For example, patients with irritable bowel syndrome (IBS) treated with PMA-ZEO showed lower blood hsCRP (highly sensitive C reactive protein) levels and decreased stool α1-anti-trypsin levels, suggesting that PMA-ZEO might decrease systemic inflammation in humans. In the same study, the corresponding increase in the immune-modulating species Bifidobacteria and *Lactobacillus* and the reduction of Firmicutes also indicate an inflammation ameliorating effect and a possible mucous layer strengthening effect (Petkov et al., 2021).

Moreover, the observed local immune-modulatory effects of clinoptilolite might involve the induction of natural immune responses through Peyers patches in the intestine and/or possible positive effects on the microbial intestinal populations through still unknown mechanism: these local effects may have a systemic “echo” on the whole immune status as well, as observed in some studies. Furthermore, the local effect in the gastrointestinal tract might underlie the whole strengthening of the immune system (Kraljevic Pavelic et al., 2018).

Stimulation of the immune response in both humans and animals by clinoptilolite may additionally be promoted by peritoneal macrophages (Pavelic et al., 2002) The clinoptilolite materials may induce a local inflammatory reaction similar to other silicate materials. This causes the attraction of peritoneal macrophages at the site and their release of TNFα, which stimulates T-cells. NFκB also acts as a regulator of targeted genes that induces T-cell activation, and this type of positive regulatory loop can amplify and perpetuate the observed local inflammatory response, which also has an immunostimulant outcome with an immunostimulatory effect by induction of IgA. Moreover, in humans, a study with immunocompromised patients showed that supplementation with clinoptilolite increases the levels of CD4^+^ and CD19^+^ and blood lymphocyte counts over a period of 6–8 weeks (Eisenwagen, 2020).

Persistent chronic inflammation status as well as a decreased immune response capacity might facilitate carcinogenesis, tumor progression, and cancer recurrence (Coussens and Werb, 2002). Currently, cancer patients undergo chemotherapy treatments that show short-term control of metastatic disease and reduce cancer-related relapse risk in an adjuvant setting, particularly in the first year after surgery when this risk is increased (You et al., 2011; Broadbridge et al., 2013). However, despite these beneficial effects of chemotherapy and oxaliplatin in particular, several side effects have been reported. The most frequent among them is CIPN, which is characterized by sensory symptoms such as pain, paresthesia, and dysesthesia. Moreover, growing evidence suggests that oxaliplatin treatment induces neurotoxic effects on the central nervous system, resulting in a deterioration of cognitive and motor functions of patients. Among the proposed mechanism of action, oxaliplatin might induce central neurotoxicity through alteration of tight junctions and the Adherens junction proteins localized on the endothelial cells of the blood–brain barrier (Branca et al., 2018). Moreover, oxaliplatin could mediate neurotoxic effects through the induction of oxidative-stress damage that causes protein oxidation, lipoperoxidation, and oxidative damage at the DNA level, as already observed in a rat model of painful oxaliplatin-induced neuropathy (Di Cesare Mannelli et al., 2012). A recent review article by researchers at Harvard Medical School showed the importance of the gut and gut bacteria in cancer treatment. A lot of cancer clinical trials are currently exploring the influence of the microbiome to help address some of the limitations and gaps in understanding. These include trials of fecal microbial transplantation, dietary supplements, and novel drugs that may influence microbiota composition (Liu and Shah, 2022; Park et al., 2022) There is growing evidence that the destruction of the protective barrier of the intestinal mucosa plays a role in the development of colorectal cancer. The assumption is that inflammation and oxidative stress have an influence on colorectal carcinogenesis. The destruction of the intestinal wall results in the influx of toxins, which promote inflammatory processes and the release of reactive oxygen species (ROS). For example, when the microbiome invades the epithelium of the intestine, inflammation and oxidative stress can promote the translocation of species through the colonic lumen causing immune response (Genua et al., 2021).

Based on these considerations, the anti-inflammatory and immunostimulatory effects of PMA-ZEO might potentially be exploited as a valid long-term therapeutic strategy for improved patient survival and quality of life.

This hypothesis is likely to be based on the statistically significant OS result for the 7-month PMA-ZEO intake period (*p* = 0.004), a borderline significant PFS result (*p* = 0.05) and relevant trends for OS after stratification of the groups over the entire follow-up duration.

These results point to PMA-ZEO protective effects, particularly underlying the better survival of patients. The current study provides important data on the beneficial effects of PMA-ZEO adjuvant supplementation for cancer patients and safety in standard oncology regimens. However, additional studies designed to study the impact of PMA-ZEO on OS, especially in the adjuvant setting, are desirable. Considering that the risk of relapse is highest in the first post-surgery years and gradually decreases over the next 10 years, enrolled patients might be followed-up for another 30 months up to 5–10 years.

Moreover, deeper knowledge and understanding of the long-term cause-and-effect relationship between the application of PMA-ZEO and reduction of local inflammation or strengthening of the immune system might be obtained in further studies. For example, such studies might be designed to monitor patients over a longer PMA-ZEO application period of *≥*7 months. We might also emphasize that the power analysis within the study presented herein showed that the number of patients was lower than the required number for conclusive statistical data. However, a positive effect on OS in first-line chemotherapy patients points to an added value of PMA-ZEO supplementation in the overall treatment outcome.

Finally, the data presented within this publication are for the PMA-ZEO material, a natural clinoptilolite processed with a specific micronization technology (Panaceo-Micro-Activation—PMA, patent WO 2018/100178A1) that changes the material´s biophysical properties in comparison with other clinoptilolite materials. Therefore, these findings cannot be generalized to other natural zeolite-clinoptilolites.

## Conclusion

Based on this 30-month follow-up analysis, positive conclusions on the PMA-ZEO clinical applications in oncology patients can be drawn. However, further studies involving a larger number of patients and designed over a longer supplementation and observational period are desirable to derive conclusive data on the quality of life and overall survival of cancer patients.

Importantly, PMA-ZEO did not negatively interfere with existing, standard chemotherapy treatment in a simultaneous regimen and appropriate time intervals. Oral PMA-ZEO application had a beneficial effect on cancer patient standard therapy and improved OS in chemotherapy patients with colorectal cancer.

This conclusion is based on the positive trends for OS regarding age, comorbidities, and oxaliplatin dose (cycles) over the whole period of the follow-up (7 months of PMA-ZEO intake and 30 months of follow-up), particularly for younger and elderly first**-**line chemotherapy patients in comparison with the placebo group. Statistically significant beneficial effects on OS were observed in the first**-**line chemotherapy patients (*p* = 0.004), and borderline significant effects on PFS (*p* = 0,05) were observed at 7 months of PMA-ZEO supplementation.

In conclusion, the data presented herein provide proof of the efficacy and safety of PMA-ZEO in a randomized controlled trial with colon cancer patients, and indicate that chemotherapy patients may benefit from an adjuvant therapy with appropriate zeolite products (e.g., PMA-zeolite).

## Data Availability

The raw data supporting the conclusion of this article will be made available by the authors, without undue reservation.
